# Efficacy, safety, and costs of nine Chinese patent medicines for the treatment of angina pectoris in CHD

**DOI:** 10.3389/fpubh.2025.1696072

**Published:** 2026-01-14

**Authors:** Jiahui Sun, Ruixuan Li, Zhenxin Wang, Tianyi Ying, Ming Hu, Naitong Zhou, Nan Yang

**Affiliations:** 1 Pharmaceutical Policy & Pharmacoeconomics Research Center, West China College of Pharmacy, Sichuan University, Chengdu, China; 2 City University of Hong Kong, Kowloon, Hong Kong SAR, China; 3 Key Laboratory of Drug-Targeting and Drug Delivery System of the Education Ministry and Sichuan Province, Sichuan Engineering Laboratory for Plant-Sourced Drug, West China School of Pharmacy, Sichuan University, Chengdu, China

**Keywords:** angina pectoris (AP), Chinese patent medicine (CPM), Cost-Consequence Analysis (CCA), network meta-analysis (NMA), Qi deficiency and blood stasis

## Abstract

**Objective:**

This study employed a multidimensional evaluation framework (of efficacy, safety, and costs) to comprehensively assess the value of Chinese patent medicines (CPMs) combined with Western medicine in treating coronary heart disease (CHD) angina pectoris with Qi deficiency and blood stasis pattern.

**Methods:**

Efficacy analysis was based on a network meta-analysis (NMA) of 24 randomized controlled trials (RCTs). Safety was assessed using a standardized framework that graded adverse events via CTCAE v5.0 and synthesized risk profiles into a Composite Safety Score. A pragmatic Cost-Consequence Analysis (CCA) was employed to synthesize direct daily treatment costs with clinical efficacy rankings (SUCRA).

**Results:**

The network meta-analysis of 24 RCTs (*n* = 2,382) showed that CPMs combined with conventional medicine significantly improved clinical efficacy (OR 3.08, 95% CI: 2.46–3.85). Dengzhan Shengmai Capsule (SUCRA 88.99%) and Shexiang Tongxin Dropping Pill (SUCRA 75.12%) ranked highest in efficacy. Safety analysis using the Composite Safety Score (CSS) identified Yangxinshi Tablet as having the most favorable profile (CSS = 3), whereas Xintong Granule and Qishen Capsule presented higher risks (CSS = 9) due to hepatotoxicity concerns or data gaps. Cost-consequence analysis revealed distinct value profiles: Yangxinshi Tablet offered the lowest daily cost (CNY 6.24) suitable for cost-minimization; Shexiang Tongxin Dropping Pill (CNY 7.28) provided a balanced cost-efficacy ratio; while Dengzhan Shengmai Capsule (CNY 10.86) represented a premium high-efficacy option. Sensitivity analysis confirmed the robustness of efficacy findings but highlighted cost fluctuations in flexible-dose regimens.

**Conclusion:**

Specific CPMs, notably Dengzhan Shengmai Capsule (maximal efficacy), Shexiang Tongxin Dropping Pill (balanced value), and Yangxinshi Tablet (cost-minimization), demonstrate significant therapeutic and economic advantages as add-on therapies. Beyond these clinical findings, this study establishes a multidimensional framework aligned with international regulatory standards, serving as a model for the future pharmacoeconomic integration of TCM into global cardiovascular care.

## 1 Introduction

Coronary Atherosclerotic Heart Disease (CHD) is a major cardiovascular disease threatening human health, caused by myocardial ischemia, hypoxia, or necrosis resulting from coronary atherosclerotic stenosis, spasm, or occlusion. According to the China Cardiovascular Health and Disease Report 2023, China has approximately 330 million cardiovascular disease (CVD) patients, including about 11.39 million CHD cases. CVD mortality ranks first (exceeding 40% of total deaths), with urban and rural CHD mortality rates in 2021 being 135.08 and 148.19 per 100,000, respectively, with higher rates among males than females. This substantial disease burden underscores the urgent need for effective management strategies for CHD, particularly for its common manifestation, angina pectoris.

Current treatments for angina pectoris in CHD primarily rely on Western medicine (e.g., nitrates and beta-blockers for symptom relief, antiplatelet drugs for prognosis improvement) and surgical interventions, such as percutaneous coronary intervention (PCI) and coronary artery bypass grafting (CABG) (1–5). While effective, these approaches may have limitations in terms of side effects, long-term efficacy, or accessibility for certain patient populations. In China, Traditional Chinese Medicine (TCM) compound preparations, particularly Chinese patent medicines (CPMs), demonstrate unique advantages due to their multi-target mechanisms (e.g., “promoting blood circulation, resolving stasis, and unblocking collaterals”).

Chinese Patent Medicines (CPMs) represent a modernized form of Traditional Chinese Medicine. It is important to distinguish Chinese Patent Medicines (CPMs) from traditional herbal decoctions. CPMs are standardized, industrially manufactured pharmaceutical products developed from TCM theories and formulas. They undergo rigorous quality control, hold market authorization from the National Medical Products Administration (NMPA), and are typically administered in fixed-dose forms such as capsules, tablets, or dripping pills. This contrasts with traditional herbal decoctions, which are customized formulations of raw herbs prepared individually for a patient based on syndrome differentiation. These products are subject to rigorous registration and approval by the National Medical Products Administration (NMPA), the primary pharmaceutical regulatory authority in China. To encourage innovation and ensure quality, high-value CPMs are often granted a “market protection period” under the Regulations on Protection of Traditional Chinese Medicines. This designation provides manufacturers with exclusive production rights for a specific duration (typically ranging from 7 to 30 years, depending on the protection grade), analogous to market exclusivity for patented drugs in Western regulatory frameworks. Clinical studies confirm that TCM preparations such as Tongxinluo Capsule can reduce angina attacks, improve electrocardiogram (ECG) results, enhance quality of life, and exhibit favorable safety profiles with economic burden and value proposition (6).

Despite the potential benefits and increasing clinical utilization of CPMs, their integration into standardized treatment paradigms faces significant challenges, primarily stemming from gaps in the existing evidence base. Firstly, the evaluation system for CPMs remains incomplete. Most existing research focuses predominantly on single dimensions, such as efficacy or safety, lacking comprehensive assessments that integrate multiple critical domains including efficacy, safety, and economy. This fragmented approach limits a holistic understanding of their clinical value. Secondly, the evidence grading for CPMs is limited. International guidelines often assign TCM lower recommendation levels (e.g., Class IIb), reflecting insufficient standardization in syndrome differentiation and a scarcity of high-quality randomized controlled trials (RCTs). Thirdly, there is an urgent need for methodological standardization. Deeper integration with modern medical research is required to advance pharmacological mechanism studies and establish universally accepted, standardized syndrome differentiation criteria and treatment protocols (7, 8).

A cornerstone of TCM practice is syndrome differentiation, which tailors treatments to specific patient patterns. This study focuses on the prevalent pattern in CHD angina known as *Qi* deficiency and blood stasis. In TCM theory, *Qi* represents vital energy that motivates physiological functions. *Qi* deficiency manifests as fatigue, shortness of breath, and spontaneous sweating. Blood stasis refers to impaired blood circulation, presenting as fixed, stabbing chest pain, a dark purplish tongue, and a choppy pulse. This pattern closely parallels the modern medical understanding of CHD, involving myocardial energy metabolic dysfunction (*Qi* deficiency) and impaired coronary microcirculation, thrombosis, or endothelial dysfunction (blood stasis).

Network meta-analyses (NMAs) have been increasingly applied in TCM research to comparatively evaluate multiple interventions, as seen in studies by Gao et al. (6) on CPMs for phlegm-blood stasis syndrome angina (7) and Zheng et al. (8) on TCM injections for heart failure. However, these prior analyses often remain confined to efficacy comparisons and frequently do not incorporate concurrent, systematic evaluations of safety and economic aspects within a unified framework.

Specifically, there is a notable scarcity of previous research that systematically and comprehensively evaluates the efficacy, safety, and economy of marketed CPMs specifically for treating angina pectoris in CHD using a multidimensional approach. This critical gap hinders the ability of clinicians and healthcare policymakers to make fully informed decisions regarding the optimal use of these medicines, balancing their therapeutic benefits against potential risks and costs. Therefore, this study aims to bridge this gap by establishing a multidimensional evaluation framework. It will systematically analyze and compare the efficacy, safety, and economic profiles of nine commonly used and insurance-listed CPMs for CHD angina with *Qi* deficiency and blood stasis pattern.

By synthesizing evidence through multidimensional framework, this research seeks to provide a comprehensive assessment of value, ultimately generating robust reference evidence to guide rational clinical decision-making and inform policies related to drug access and reimbursement.

## 2 Methods

### 2.1 Methods for efficacy analysis

#### 2.1.1 Overall design and data sources

This study is a systematic review and meta-analysis based on 24 randomized controlled trials (RCTs) (9–32). The included trials compared the efficacy of a combination regimen (one of nine specific CPMs plus conventional medicine) against conventional medicine alone for treating angina pectoris in coronary heart disease patients with Qi deficiency and blood stasis syndrome. The primary outcome was dichotomous (clinically effective or not). To provide a comprehensive efficacy evaluation, we employed a dual analytical strategy: initial pairwise meta-analyses for each CPM vs. control, followed by a network meta-analysis (NMA) to compare all interventions within an integrated framework.

Crucially, we strictly distinguished the data sources used for the different dimensions of this evaluation to ensure methodological rigor. The assessment of clinical efficacy and safety was based exclusively on the aforementioned RCTs to guarantee internal validity, explicitly excluding any observational or non-randomized “real-world studies” from the network meta-analysis. In contrast, the assessment of economy and accessibility relied on distinct Real-World Data (RWD) sources, specifically provincial drug procurement platforms and hospital information systems, to reflect actual market conditions.

#### 2.1.2 Search strategy and study selection

We conducted a network meta-analysis by systematically searching PubMed, Embase, CNKI, VIP, and Wanfang databases for randomized controlled trials (RCTs) on Chinese patent medicines (CPMs) treating angina symptoms (chest pain, tightness, dyspnea, palpitations) in coronary heart disease with Qi deficiency-blood stasis. After screening and evaluation, eligible studies were selected, data extracted, and quantitative synthesis performed on clinical efficacy rates. The search strategy used Boolean operators (“*Qi* deficiency and blood stasis” AND “coronary heart disease (CHD)” AND “angina pectoris”) targeting titles/abstracts/keywords, with inclusion criteria prioritizing CPM-Western combination therapies (Table 1).

**Table 1:** Inclusion and exclusion criteria.

**Category**	**Inclusion criteria**	**Exclusion criteria**
Reason 1: Study Population	Studies without demographic or clinical selection criteria (gender, age, health status).	Studies exclusively targeting specific subgroups (e.g., males, females, older adult(s), post-PCI patients) were excluded to maintain baseline comparability and minimize clinical heterogeneity across the network.
Reason 2: Interventions	Observation group: Combination therapy with Chinese patent medicines (CPMs) and conventional medicine (e.g., nitrates, β-blockers, guideline-recommended agents). Control group: Conventional medicine alone.	• Monotherapy (CPMs alone, Western medicine alone, or traditional herbal decoctions alone). • Combined use of traditional Chinese medicine (herbal decoctions, acupuncture, etc.) with Western medicine. • Mixed-intervention studies without clear distinction between combination and monotherapy groups.
Reason 3: Outcome Measures	Primary endpoint: Clinical effective rate for angina symptoms (effective cases/total cases), validated by authoritative guidelines.	• Non-standard Primary Endpoint: Studies that did not utilize “clinical effective rate” (based on angina symptom reduction) as a primary efficacy endpoint. • Incompatible Data Format: Studies reporting outcomes solely as continuous variables (e.g., Seattle Angina Questionnaire scores, VAS pain scores, or mean frequency of angina attacks) without providing the corresponding number of responders. • Unconvertible Data: Studies lacking extractable dichotomous data (i.e., the specific count of effective/ineffective cases) according to the pre-defined efficacy criteria (reduction ≥50% or ≥80%), as these cannot be accurately derived from aggregate continuous data (Mean ± SD) without individual patient data.
Reason 4: Drug Status	CPMs and Western drugs must hold valid NMPA marketing authorization. Drugs must remain marketing available (verified via NMPA database and manufacturer announcements) at the data collection timepoint.	• Drugs withdrawn from the market (voluntary or regulatory discontinuation)

The primary efficacy endpoint was the number of patients achieving clinical effectiveness (Total Clinical Effective Rate). This is a dichotomous outcome derived from the Guiding Principles for Clinical Research of New Chinese Medicines. “Clinical effectiveness” was defined as a strictly measured composite endpoint. A patient was classified as a “responder” (effective case) if they met the criteria for either “Marked Effect” (symptoms such as angina abolished or reduced by ≥80%) or “Effective” (symptoms reduced by 50%−80%). Patients failing to meet these thresholds were classified as “Ineffective.”

Following a systematic literature search and screening against the inclusion criteria of the National Basic Medical Insurance, Work Injury Insurance, and Maternity Insurance Drug List (2024 Edition) issued by the National Healthcare Security Administration (NHSA), a total of **nine** Chinese patent medicines (CPMs) were ultimately selected for inclusion in this study (Table 2).

**Table 2:** List of included medications.

**Medication name**	**National insurance category**	**Main ingredients**
Dengzhan Shengmai Capsule	Category B	*Erigeron breviscapus* (Deng Zhan Xi Xin), *Radix et Rhizoma Ginseng* (Ren Shen), *Fructus Schisandrae Chinensis* (Wu Wei Zi), *Radix Ophiopogonis* (Mai Dong).
Fufang Dilong Tablet	Category B	*Pheretima* (Di Long), *Rhizoma Chuanxiong* (Chuan Xiong), *Radix Astragali* (Huang Qi), *Radix Achyranthis Bidentatae* (Niu Xi).
Naoxin Tong Capsule	Category B	*Radix Astragali* (Huang Qi), *Hirudo* (Shui Zhi), *Scorpio* (Quan Xie), *Pheretima* (Di Long), *Radix et Rhizoma Salviae Miltiorrhizae* (Dan Shen), *Radix Angelicae Sinensis* (Dang Gui), *Rhizoma Chuanxiong* (Chuan Xiong), *Radix Paeoniae Rubra* (Chi Shao).
Qishen Yiqi Dropping Pill	Category B	*Radix Astragali* (Huang Qi), *Radix et Rhizoma Salviae Miltiorrhizae* (Dan Shen), *Radix et Rhizoma Notoginseng* (San Qi), *Lignum Dalbergiae Odoriferae* (Jiang Xiang).
Shexiang Tongxin Dropping Pill	Category B	*Moschus* (She Xiang), *Radix et Rhizoma Ginseng* (Ren Shen), *Radix et Rhizoma Salviae Miltiorrhizae* (Dan Shen), *Venenum Bufonis* (Chan Su), *Calculus Bovis Artifactus* (Ren Gong Niu Huang), *Borneolum Syntheticum* (Bing Pian).
Tongxinluo Capsule	Category A	*Radix et Rhizoma Ginseng* (Ren Shen), *Hirudo* (Shui Zhi), *Scorpio* (Quan Xie), *Radix Paeoniae Rubra* (Chi Shao), *Periostracum Cicadae* (Chan Tui), *Eupolyphaga Steleophaga* (Tu Bie Chong), *Scolopendra* (Wu Gong), *Lignum Santali Albi* (Tan Xiang), *Lignum Dalbergiae Odoriferae* (Jiang Xiang), *Olibanum* (Ru Xiang), *Semen Ziziphi Spinosae* (Suan Zao Ren), *Borneolum Syntheticum* (Bing Pian).
Yangxinshi Tablet	Category B	*Radix Astragali* (Huang Qi), *Radix Codonopsis* (Dang Shen), *Radix et Rhizoma Salviae Miltiorrhizae* (Dan Shen), *Radix Puerariae Lobatae* (Ge Gen), *Herba Epimedii* (Yin Yang Huo), *Fructus Crataegi* (Shan Zha), *Rhizoma Corydalis* (Yan Hu Suo).
Xintong Granule	Category B	*Radix Astragali* (Huang Qi), *Radix Codonopsis* (Dang Shen), *Radix Ophiopogonis* (Mai Dong), *Radix et Rhizoma Salviae Miltiorrhizae* (Dan Shen), *Radix et Rhizoma Notoginseng* (San Qi), *Radix Puerariae Lobatae* (Ge Gen), *Rhizoma Corydalis* (Yan Hu Suo), *Thallus Laminariae* (Kun Bu).
Qishen Capsule	Category B	*Radix Astragali* (Huang Qi), *Radix et Rhizoma Salviae Miltiorrhizae* (Dan Shen), *Radix et Rhizoma Notoginseng* (San Qi), *Lignum Dalbergiae Odoriferae* (Jiang Xiang).

Latin names are standardized based on the Chinese Pharmacopeia. Category A represents drugs fully covered by the National Basic Medical Insurance; Category B represents drugs partially covered (typically requiring a co-payment of 10–20%).

**Figure 1:**
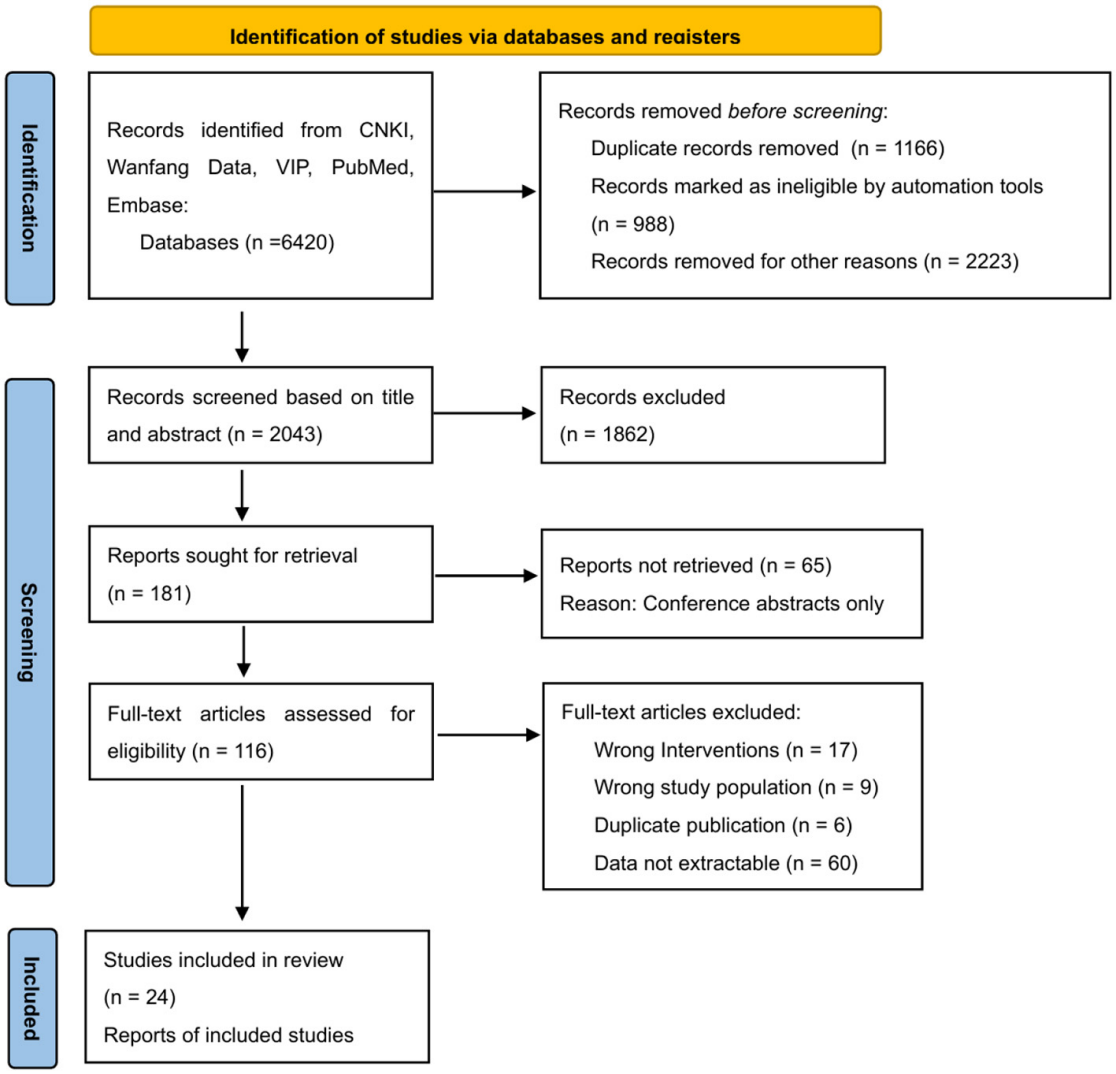
PRISMA flow diagram of literature screening and inclusion from Chinese databases.

A detailed flow diagram of the literature screening process for the network meta-analysis is presented in Figure 1, adhering to PRISMA (Preferred Reporting Items for Systematic Reviews and Meta-Analyses) guidelines. This dual approach ensured methodological rigor and minimized selection bias in evidence synthesis.

#### 2.1.3 Quality assessment

Study characteristics, patient demographics, intervention details, outcome data, and safety information were extracted from the included RCTs by two independent reviewers using a standardized form. Any discrepancies were resolved through discussion or adjudication by a third reviewer.

The methodological quality of each included RCT was independently assessed by two reviewers using the Cochrane Risk of Bias tool (ROB 2.0). This tool evaluates five domains: (1) the randomization process, (2) deviations from intended interventions, (3) missing outcome data, (4) measurement of the outcome, and (5) selection of the reported result. Each domain and the overall study were judged as having a “low risk,” “some concerns,” or “high risk” of bias.

#### 2.1.4 Statistical analysis

**1) Analytical Software:** All statistical analyses were performed using StataMP-64 (Version 15.0, StataCorp, College Station, TX) and R software (Version 4.3.1, The R Foundation for Statistical Computing). Stata was used for conducting pairwise and network meta-analyses, generating forest plots, and performing sensitivity analyses. R was utilized for generating funnel plots and additional visualizations.

**2) Pairwise meta-analysis:** To assess the specific efficacy of each CPM against the common control, a pairwise meta-analysis stratified by CPM type was first conducted. Given the negligible statistical heterogeneity observed, the Mantel-Haenszel fixed-effect model was applied. Results are presented as odds ratios (ORs) with 95% confidence intervals (CIs).

**3) Network meta-analysis (NMA):** Subsequently, a frequentist network meta-analysis was performed within a fixed-effect framework to integrate direct and indirect evidence and compare all interventions simultaneously. The Surface Under the Cumulative Ranking Curve (SUCRA) was calculated to estimate the probability of each intervention being the most effective and to establish a treatment hierarchy.

**4) Assessment of heterogeneity and inconsistency:** Statistical heterogeneity within each pairwise comparison was assessed using Cochran's *Q* test and quantified with the *I*^2^ statistic. Global network heterogeneity was also examined. Assessment of inconsistency between direct and indirect evidence was not applicable as the network structure was star-shaped with a single common control (no closed loops).

**5) Assessment of publication bias:** For the main efficacy comparison, potential publication bias was evaluated through visual inspection of funnel plots and quantified using Egger's linear regression test (*p*-value < 0.05 indicating significant asymmetry). The Duval and Tweedie non-parametric “Trim-and-Fill” method was applied to estimate and adjust for potentially missing studies.

**6) Sensitivity analysis:** To test the robustness of the primary pooled efficacy estimate, a leave-one-out sensitivity analysis was conducted. This was implemented under a random-effects model in Stata, involving the iterative removal of each individual study and recalculation of the overall OR and its 95% CI to identify any disproportionately influential studies.

#### 2.1.5 Certainty of evidence

The overall certainty (quality) of the evidence for the primary outcome from the network meta-analysis was evaluated using the GRADE framework for NMA. The certainty (rated as high, moderate, low, or very low) for each comparison against conventional medicine was assessed by considering the following domains: risk of bias, inconsistency (statistical heterogeneity), indirectness, imprecision, and publication bias. The evidence was downgraded by one or two levels for serious or very serious concerns in any domain.

**1) Analytical software and tools**

Statistical analyses and data visualization were performed using a combination of StataMP-64 (Version 15.0, StataCorp, College Station, TX) and R software (Version 4.3.1, The R Foundation for Statistical Computing). The Cochrane Risk of Bias 2.0 (RoB 2) tool was employed for quality assessment.

**2) Pairwise meta-analysis and network visualization**

Stata was utilized to conduct pairwise meta-analyses and generate the associated forest plots. Results were expressed as odds ratios (ORs) with 95% confidence intervals (CIs). Statistical heterogeneity was assessed using Cochran's Q test and quantified with the I2I2 statistic. Additionally, the network geometry was visualized using Stata to generate the Network Plot, illustrating the distribution of evidence.

**3) Bayesian network meta-analysis**

To integrate direct and indirect evidence, a Bayesian network meta-analysis (NMA) was performed using the gemtc package in R. The analysis employed Markov Chain Monte Carlo (MCMC) simulation.

- **Model specifications:** We ran four parallel MCMC chains. Technical details regarding the Bayesian model priors—specifically the standard deviation of random effects (sd.d) and initial values for relative treatment effects (d.0.x)—are explicitly defined in Supplementary Appendix A.
- **Convergence diagnostics:** Convergence of the MCMC chains was assessed using the Brooks-Gelman-Rubin diagnostic (Potential Scale Reduction Factor, PSRF) and trace plots.
- **Ranking outputs:** The probabilistic hierarchy of interventions was visualized using R to generate the League Table and the Ranking Probability Histogram. The Surface Under the Cumulative Ranking Curve (SUCRA) was calculated to estimate the comparative efficacy ranking.

**4) Assessment of inconsistency and bias**

Assessment of inconsistency between direct and indirect evidence was not applicable as the network structure was star-shaped (a single common control with no closed loops). Potential publication bias was evaluated through visual inspection of funnel plots generated using R software, and asymmetry was further quantified using Egger's linear regression test.

**5) Sensitivity analysis**

To assess the robustness of the primary findings, a leave-one-out sensitivity analysis was conducted using Stata. This involved the iterative removal of individual studies and recalculation of the pooled estimates to generate sensitivity plots and identify any disproportionately influential studies.

### 2.2 Methods for safety analysis

Safety assessments integrated data compilation with standardized analytical frameworks to ensure both transparency and comparability. The complete safety profiles of the nine included Chinese patent medicines (CPMs)—encompassing all documented adverse reactions, contraindications, and precautions—were first compiled from the clinical studies included in the network meta-analysis and from official drug package inserts. This comprehensive dataset is presented in Table 3.

**Table 3:** Summary of medication safety information.

**Medication name**	**Adverse reactions**	**Contraindications**	**Precautions**
Dengzhan Shengmai Capsule	1. Gastrointestinal reactions: Dry mouth, nausea, abdominal distension, diarrhea. 2. Allergic reactions: Rash, pruritus, dizziness, palpitations.	Contraindicated during the acute phase of cerebral hemorrhage.	1. Use with caution in patients allergic to this product. 2. If gastrointestinal reactions occur, take within 30 minutes after meals. 3. This product is in capsule form; do not remove the capsule shell to ingest the contents directly.
Fufang Dilong Tablet	Some patients may experience gastric discomfort 2–3 days after taking the medication.	Not suitable for individuals with heat-related syndromes, such as phlegm-heat syndrome, fire stagnation syndrome, or blood stasis-heat syndrome.	Uncertain
Naoxin Tong Capsule	Uncertain	Contraindicated for pregnant women.	Patients with gastric conditions should take after meals.
Qishen Yiqi Dropping Pill	Post-marketing surveillance data indicate the following adverse reactions: Gastrointestinal system: Nausea, vomiting, bloating, and other gastrointestinal discomfort. Skin and appendages: Rash, pruritus, flushing, and other allergic skin reactions.	Contraindicated in patients with allergic reactions to this product.	1. Use with caution in pregnant women and individuals with allergic predisposition. 2. Consult a doctor if gastrointestinal reactions occur after taking the medication.
Shexiang Tongxin Dropping Pill	In rare cases, patients may experience body heat and facial flushing after taking the medication, which resolve quickly after discontinuation. A very small number of patients may report a tingling or numbing sensation on the tongue. Higher doses may lead to elevated ALT levels.	Contraindicated in pregnant women.	1. Use with caution in patients with liver or kidney dysfunction. 2. This product contains the toxic medicinal ingredient “toad venom” (Chansu); strictly adhere to the prescribed dosage in the instructions. 3. During clinical trials, the following adverse events were observed: 1 case of moderate glaucoma with increased intraocular pressure, 1 case of mild body heat and facial flushing, and 1 case of mild epigastric distension and discomfort. All three cases resolved and were assessed as unlikely related to the investigational drug. 4. Athletes should use with caution.
Tongxinluo Capsule	Post-marketing surveillance data indicate the following gastrointestinal adverse reactions may occur: nausea, vomiting, abdominal pain, bloating, diarrhea, and gastric discomfort, as well as rash, pruritus, and dizziness.	Contraindicated in patients with hemorrhagic diseases, pregnant women, women during menstruation, and those with stroke of Yin deficiency with fire effulgence pattern.	Patients experiencing gastric discomfort after taking the medication should switch to taking it after meals.
Yangxinshi Tablet	A very small number of patients may experience gastric discomfort.	Contraindicated in pregnant women.	Patients experiencing gastric discomfort after administration should follow the doctor's advice.
Xintong Granule	Monitoring data indicate the following adverse reactions may occur with this product: 1. Hepatobiliary disorders: Liver injury. 2. Gastrointestinal disorders: Nausea, vomiting, diarrhea, abdominal pain, bloating, loss of appetite, acid reflux, heartburn, constipation, dyspepsia, etc. 3. Skin and allergic reactions: Rash, pruritus, erythema, drug eruption, maculopapular rash, hyperhidrosis, fever, flushing, edema, etc. 4. Others: Dizziness, headache, dyspnea, etc.	1. Contraindicated in pregnant women. 2. Contraindicated in patients allergic to this product or any of its components. 3. Contraindicated in patients with a history of drug-induced liver injury.	1. This product contains Polygonum multiflorum (He Shou Wu) and Epimedium (Yin Yang Huo). Liver function tests should be monitored during treatment. Discontinue use and seek medical attention if abnormalities occur. 2. Concomitant use with other hepatotoxic drugs should be avoided. 3. Use with caution in patients with a known family history of drug-induced liver injury. 4. This product contains Codonopsis (Dang Shen) and Salvia miltiorrhiza (Dan Shen), and should not be used together with Veratrum nigrum (Li Lu). 5. Patients experiencing acid reflux may take the medication after meals. 6. Avoid alcohol and spicy or irritating foods.
Qishen Capsule	Uncertain	Uncertain	1. Use with caution in pregnant women and women during menstruation. 2. Use with caution in individuals with bleeding tendencies. 3. Currently, there are no clinical trial data available for pregnant or breastfeeding women. 4. This product is hygroscopic (moisture-absorbing). Once opened, consume promptly. 5. Do not use if the physical characteristics of the product have changed.

To enable a standardized and comparative evaluation of these profiles, we applied a two-part analytical framework. First, the qualitative descriptions of adverse reactions were classified according to the Common Terminology Criteria for Adverse Events (CTCAE) v5.0 (33) classify each reported AE based on its clinical description. AE severity was graded as: Grade 1 (Mild), Grade 2 (Moderate), Grade 3 (Severe), Grade 4 (Life-threatening), or Grade 5 (Death).

Second, to synthesize the multi-faceted risk information (ADR severity, contraindication breadth, and precaution complexity) into a single comparable metric, a Composite Safety Score (CSS) was developed. The CSS is derived from three discrete domains, each scored from 1 (most favorable) to 3 (least favorable): (1) Domain A (ADR Severity): Reflects the highest CTCAE grade associated with the medication's ADR profile. (2) Domain B (Contraindication Scope): Assesses the breadth and clinical seriousness of listed contraindications. (3) Domain C (Precaution Complexity): Evaluates the operational burden and monitoring requirements outlined in precautions.

The final CSS for each CPM is the sum of these three domain scores (range: 3–9), with a lower total score indicating a more favorable overall safety profile. All drug information was verified against the NMPA database and the commercial pharmaceutical database Yaozh.com.

### 2.3 Methods for cost-consequence analysis

An economic evaluation was systematically conducted to compare the cost profiles of different Chinese patent medicines. In selecting the evaluation methodology, we acknowledge that Cost-Benefit Analysis (CBA), grounded in Welfare Economics, offers a comprehensive framework for health decision-making (34). However, CBA necessitates the complex step of monetizing health outcomes, typically through the Human Capital Approach (HCA) or Willingness-to-Pay (WTP) methods. The application of this approach in the health industry is still under development and its methodology has not yet reached a broad consensus (34, 35). Therefore, this study adopts a pragmatic Cost-Consequence Analysis (CCA) approach. We conducted a direct cost comparison and integrated this with the clinical efficacy findings. Distinct from the clinical data derived from RCTs, the economic parameters utilized real-world data. Cost parameters were sourced from NMPA registration records and real-world drug pricing data extracted from Yaozh.com, a leading Chinese pharmaceutical pricing database. Key metrics included unit price per medication and daily treatment cost, calculated based on standardized dosage regimens and course durations. These direct costs were then systematically synthesized with SUCRA (the non-monetized clinical efficacy rankings), which can provide a transparent and practical tool for decision-makers to balance therapeutic benefit against cost burden.

## 3 Results

### 3.1 Efficacy results

#### 3.1.1 Risk of bias assessment

The network meta-analysis was performed using a Random-Effects Model due to the presence of heterogeneity (heterogeneity parameter σ = 1.02, 95% CI: 1.00–1.06). Convergence diagnostics confirmed the robustness of the model, with all Potential Scale Reduction Factor (PSRF) values approaching 1.0, indicating good chain mixing (detailed diagnostic plots and parameter specifications are provided in Supplementary Appendix A).

**Figure 2:**
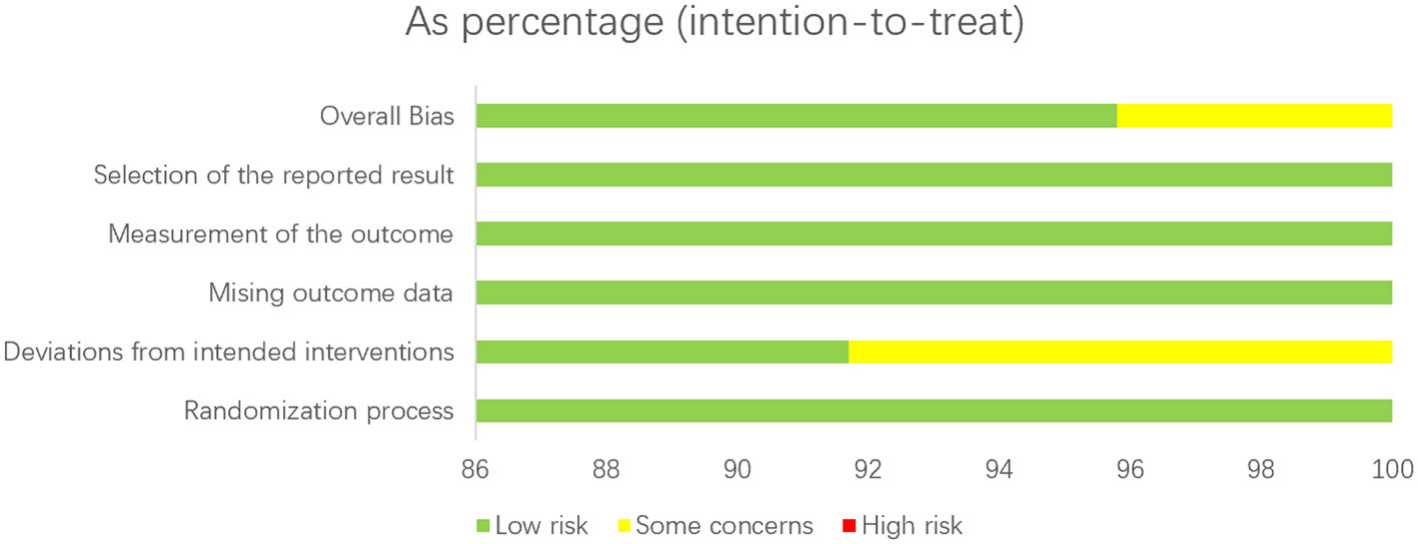
Risk of bias summary for the included randomized controlled trials (assessed with the Cochrane ROB 2.0).

The methodological quality of the 24 included randomized controlled trials (RCTs) was rigorously evaluated using the Cochrane Risk of Bias tool (ROB 2.0). The assessment revealed a generally low risk of bias across all domains. Specifically, the randomization process, missing outcome data, measurement of the outcome, and selection of the reported result were all rated as having a low risk of bias in 100% of the studies. In the domain of deviations from intended interventions, the majority of studies (91.7%) were judged as low risk, while a small proportion (8.3%) raised some concerns. Consequently, the overall risk of bias was classified as low for 95.8% of the studies (23 out of 24), with only one study (4.2%) presenting some concerns. No study was rated as having a high risk of bias in any domain (Figure 2).

#### 3.1.2 Network structure and evidence base

**Figure 3:**
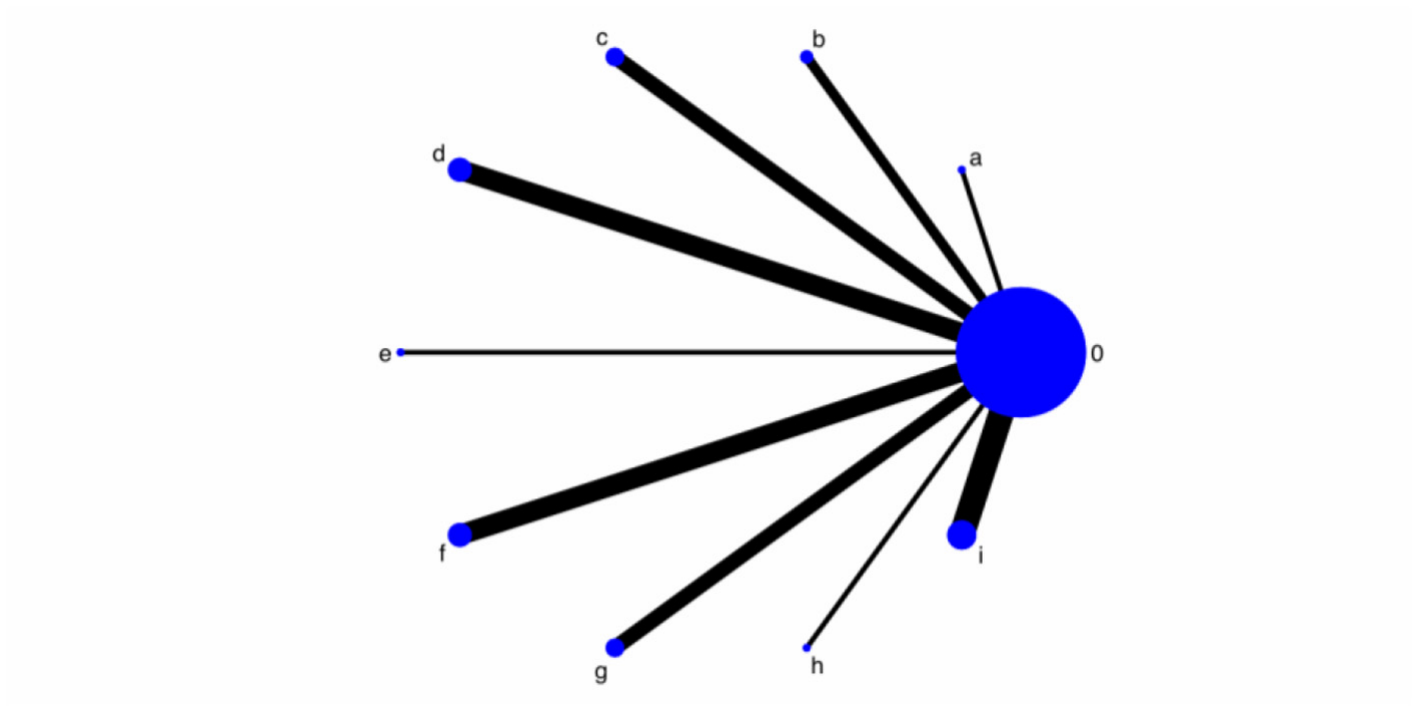
Network Plot of Interventions for AP in CHD with *Qi* Deficiency and Blood Stasis Pattern. Interventions combining CPMs with Conventional medicine are labeled by CPMs' names.

Figure 3 illustrates a star-shaped evidence network centered on conventional medicine (Node 0). All nine active interventions (Nodes a–i) were compared directly against the control, with no direct head-to-head comparisons between active treatments (i.e., no closed loops). Node sizes and line widths correspond to sample sizes and the number of studies, respectively. The thickest lines connect the control with interventions d, c, and f, indicating that these comparisons rely on the most extensive evidence. Table 4 provides the intervention codes.

**Table 4:** Intervention names and alphanumeric code reference.

**Code**	**0**	**a**	**b**	**c**	**d**	**e**	**f**	**g**	**h**	**i**
Intervention	Conventional Medicine	Dengzhan Shengmai Capsule + Conventional Medicine	Fufang Dilong Tablet + Conventional Medicine	Naoxin Tong Capsule + Conventional Medicine	Qishen Yiqi Dropping Pill + Conventional Medicine	Shexiang Tongxin Dropping Pill + Conventional Medicine	Tongxinluo Capsule + Conventional Medicine	Yangxinshi Tablet + Conventional Medicine	Xintong Granule + Conventional Medicine	Qishen Capsule + Conventional Medicine

Conventional medicine: Including Aspirin Enteric-coated Tablets, Clopidogrel Bisulfate Tablets, Atorvastatin Calcium Tablets and Isosorbide Mononitrate Sustained-release Tablets, etc.

#### 3.1.3 Pairwise meta-analysis

This meta-analysis included a total of 24 randomized controlled trials, involving 2,382 participants. Given the negligible overall heterogeneity (*I*^2^ = 0.0%, *p* = 0.956), a random-effects model was employed. The pooled analysis demonstrated a statistically significant improvement in clinical outcomes with Chinese patent medicine (CPM) treatment compared to the control, yielding an overall odds ratio (OR) of 3.08 (95% CI: 2.46–3.85, *z* = 9.80, *p* < 0.001).

Subgroup analyses stratified by specific CPM formulations revealed consistent direction of benefit. Statistically significant efficacy was observed for six of the nine CPMs: Naoxin Tong Capsule (OR = 4.63, 95% CI: 2.13–10.10), Yangxinshi Tablet (OR = 3.81, 95% CI: 1.88–7.69), Dengzhan Shengmai Capsule (OR = 3.45, 95% CI: 1.52–7.85), Tongxinluo Capsule (OR = 3.22, 95% CI: 1.64–6.29), Qishen Yiqi Dripping Pill (OR = 2.99, 95% CI: 1.83–4.88), and Qishen Capsule (OR = 2.67, 95% CI: 1.78–4.02). For the remaining three CPMs—Fufang Dilong Tablet, Shexiang Tongxin Dripping Pill, and Xintong Granule—the point estimates also favored the treatment, although the results did not reach statistical significance (*p* > 0.05), likely due to wider confidence intervals associated with fewer studies and smaller sample sizes in these subgroups.

**Figure 4:**
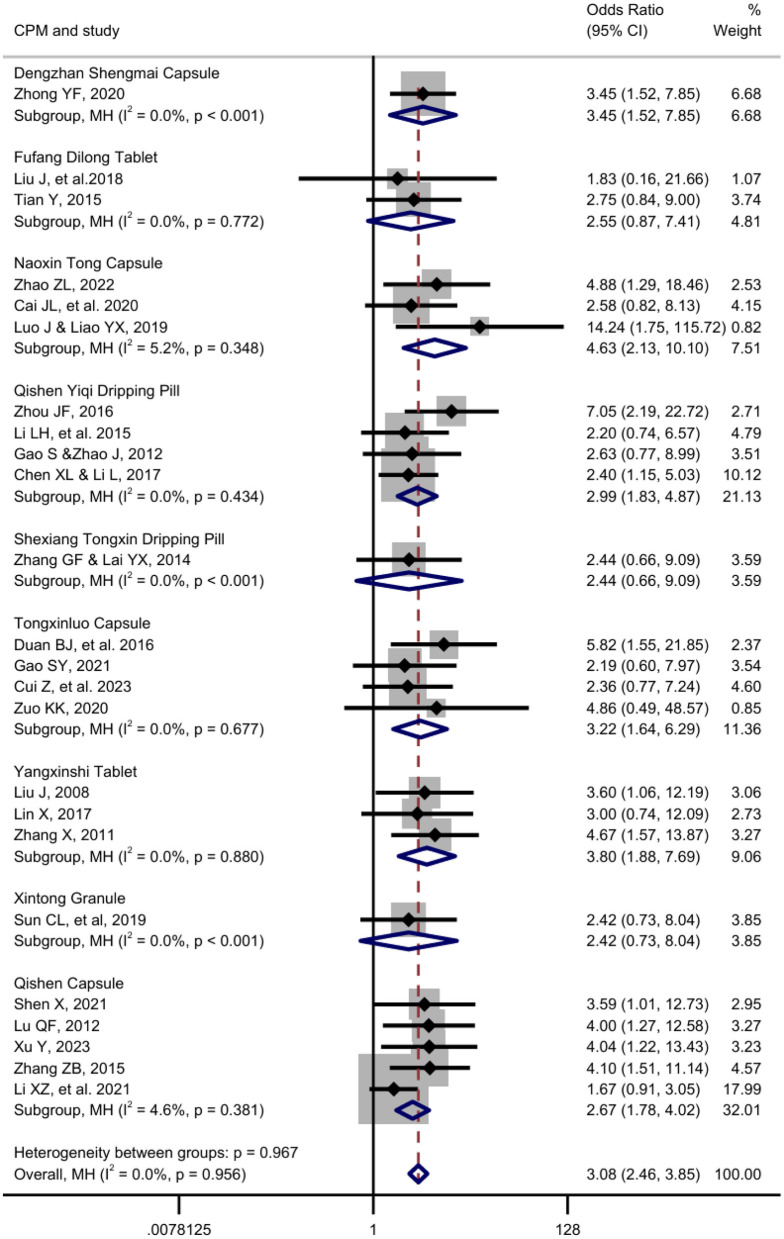
Forest plot of outcomes from different CPM interventions. The outcome measured is the number of patients achieving clinical effectiveness (a desired outcome). Therefore, an Odds Ratio (OR) > 1 indicates a beneficial effect favoring the treatment over the control. Weights and between-subgroup heterogeneity test are from Mantel-Haenszel model.

Notably, the test for differences between subgroups was not significant (*Q* = 2.37, *df* = 8, *p* = 0.967). Heterogeneity within each CPM subgroup was also minimal (I^2^ ranging from 0.0% to 5.2%), indicating a consistent treatment effect across the different formulations studied (Figure 4).

#### 3.1.4 Network meta-analysis: relative efficacy and ranking

**Figure 5:**
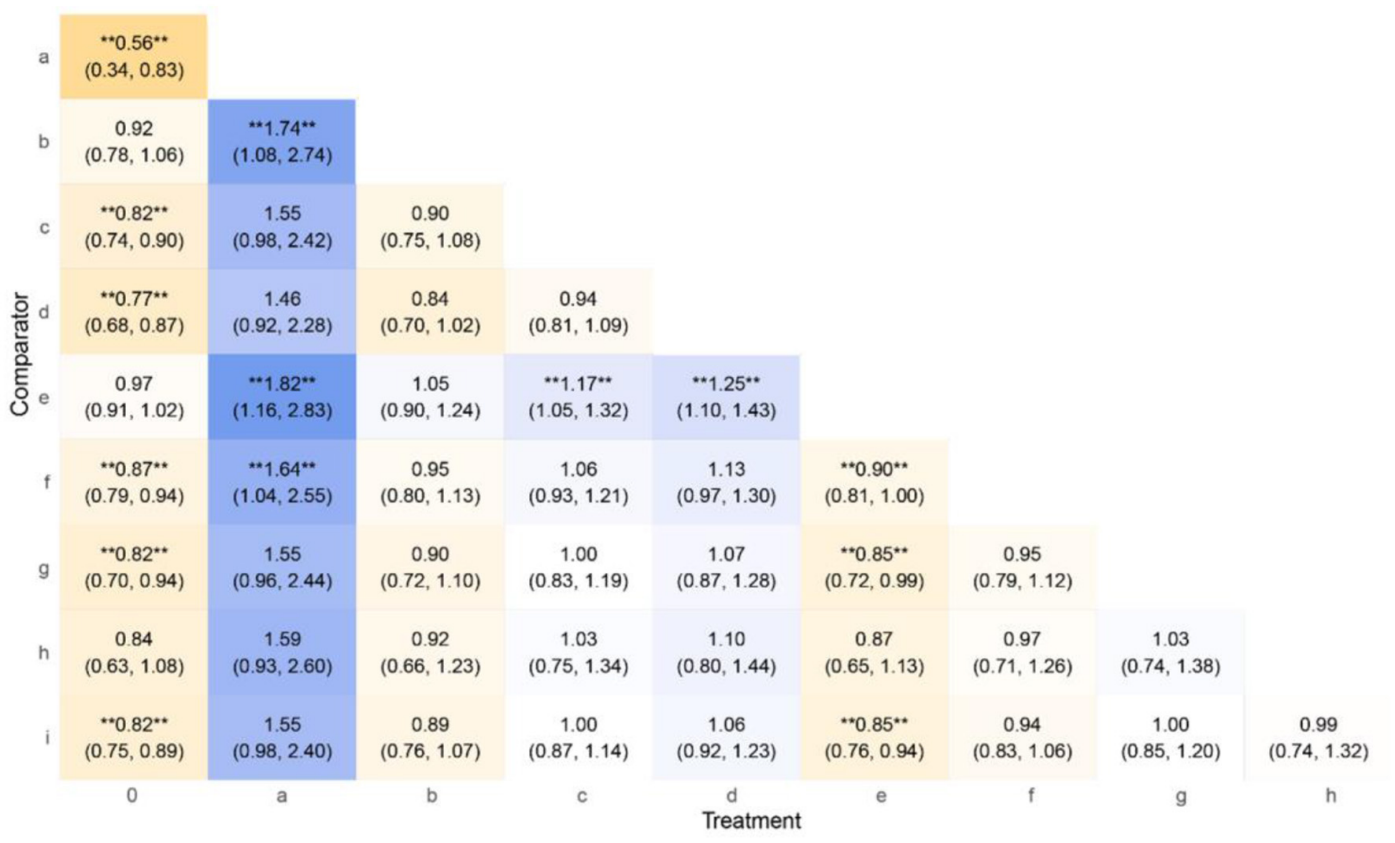
League table of interventions for angina pectoris in CHD with *Qi* deficiency and blood stasis pattern. ** Highly significant.

The significantly effective treatment groups (OR > 1 with confidence intervals excluding 1) included Group a (OR = 1.55, 95% CI: 0.98–2.42), which demonstrated a potential therapeutic advantage but required cautious interpretation due to partial overlap of the confidence interval with the null value; Group c (OR = 1.22, 95% CI: 1.11–1.35), showing stable superiority and high reliability supported by a narrow confidence interval; and Group e (OR = 1.30, 95% CI: 1.16–1.46), characterized by a robust effect size and statistical significance. Among the groups with non-significant or inverse trends, Group b (OR = 0.61, 95% CI: 0.37–0.92) exhibited a confidence interval partially overlapping the null value (OR = 1), suggesting no significant difference from the control group. In contrast, Group f (OR = 0.64, 95% CI: 0.39–0.96) displayed OR < 1 (confidence interval excluding 1), indicating potential superiority of the control group, though further clinical studies are needed to confirm this finding (Figure 5).

**Figure 6:**
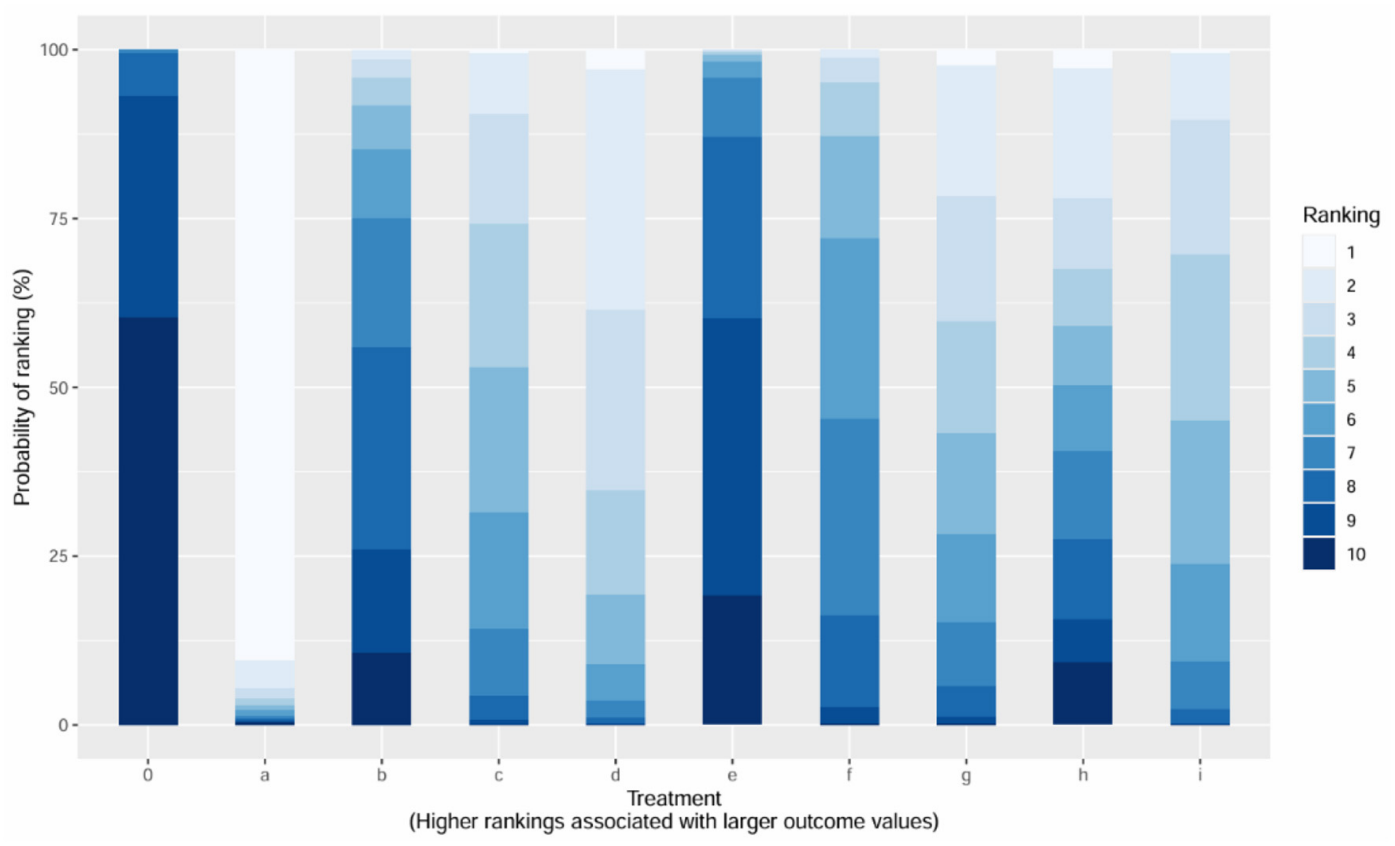
Ranking probability histogram for different interventions.

The cumulative ranking probability (SUCRA) analysis evaluated the liGranuleshood of each treatment being the optimal intervention (scale: 0–100%). Group a ranked first with a SUCRA value of 88.99%, indicating the highest probability of superiority. Group i followed with a SUCRA value of 62.35%, suggesting moderate effectiveness. In contrast, Group 0 (control) showed markedly inferior efficacy (SUCRA = 5.23%), underscoring the significant advantage of active treatment groups. Among other notable rankings, Group c (SUCRA = 58.55%) and Group e (SUCRA = 75.12%) demonstrated competitive performance, while Group b (SUCRA = 28.61%) and Group f (SUCRA = 42.30%) ranked lower, reflecting limited therapeutic benefits. These results align with the league table and forest plot findings, reinforcing the consistency of Group a and Group e as the most robust interventions (Figure 6).

#### 3.1.5 Sensitivity analysis and publication bias

To quantitatively assess the impact of potential publication bias, we conducted a Trim-and-Fill analysis. The results indicated that no studies needed to be imputed to achieve funnel plot symmetry, and the adjusted effect estimate remained identical to the original pooled estimate (logOR = 4.663). This suggests that while minor asymmetry was detected by Egger's test, the overall conclusions of our meta-analysis are robust to the potential influence of publication bias.

**Figure 7:**
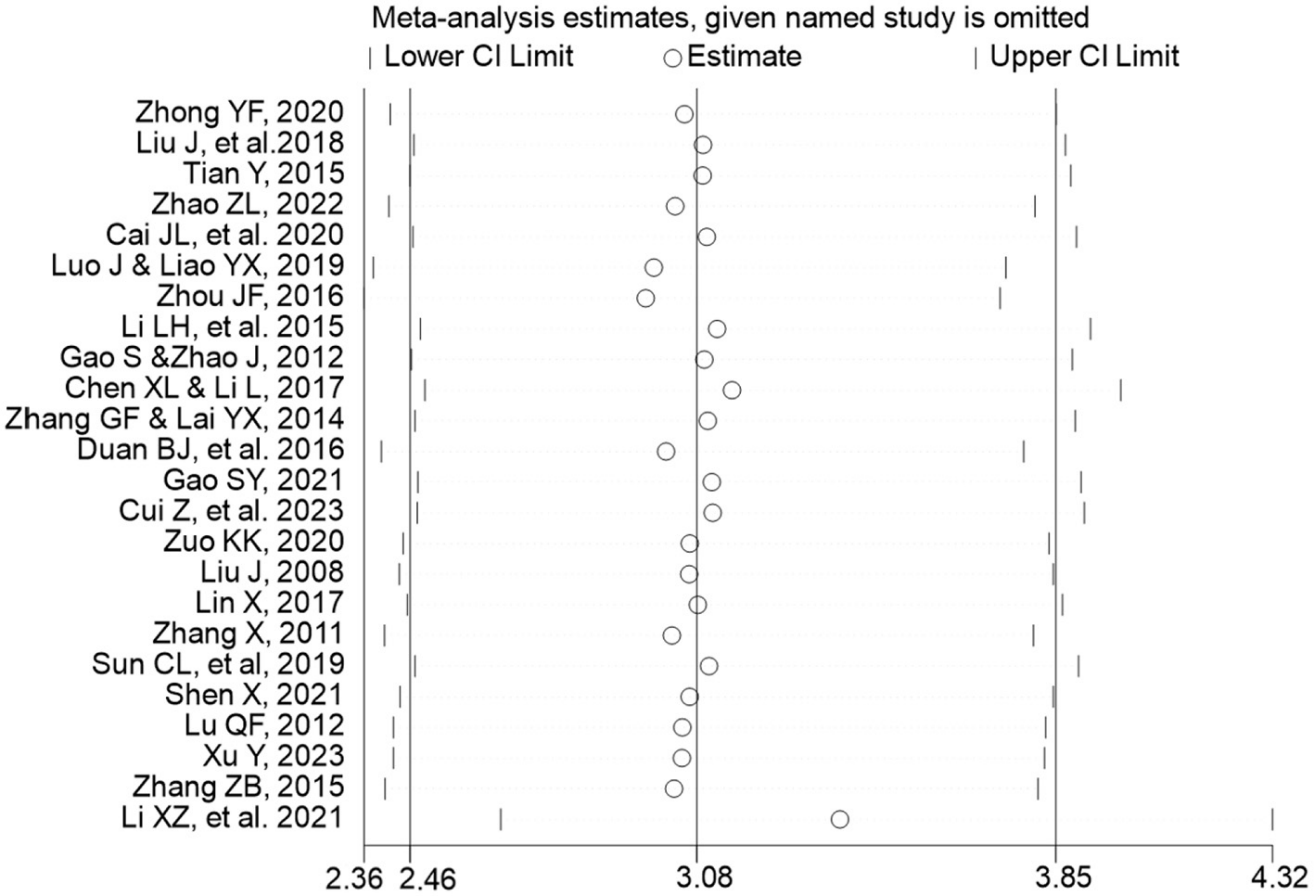
Sensitivity analysis of pooled odds ratios using the leave-one-out method (Random-Effects Model).

A sensitivity analysis was performed using a random-effects model to evaluate the robustness of the pooled results by iteratively excluding each individual study. The analysis revealed that the odds ratio (OR) estimates remained consistently between 2.98 and 3.39 after excluding any single study, with all recalculated 95% confidence intervals overlapping with the original pooled estimate (OR = 3.08, 95% CI: 2.46–3.85). Notably, the largest deviation occurred when the study by Li et al. (16) was omitted, yielding an OR of 3.39 (95% CI: 2.65–4.32), while the smallest estimate was observed after excluding Zhou (31) (OR = 2.97, 95% CI: 2.36–3.73). Despite these variations, all recalculated confidence intervals substantially overlapped with the original result, and no exclusion led to statistically significant alterations in the overall conclusion. These findings demonstrate that the results of the meta-analysis remain robust across sensitivity analyses using odds ratios, indicating that the pooled effect estimate is not unduly influenced by any single included study. The stability of the effect estimates across all iterations supports the reliability of the primary findings from the meta-analysis (Figure 7).

**Figure 8:**
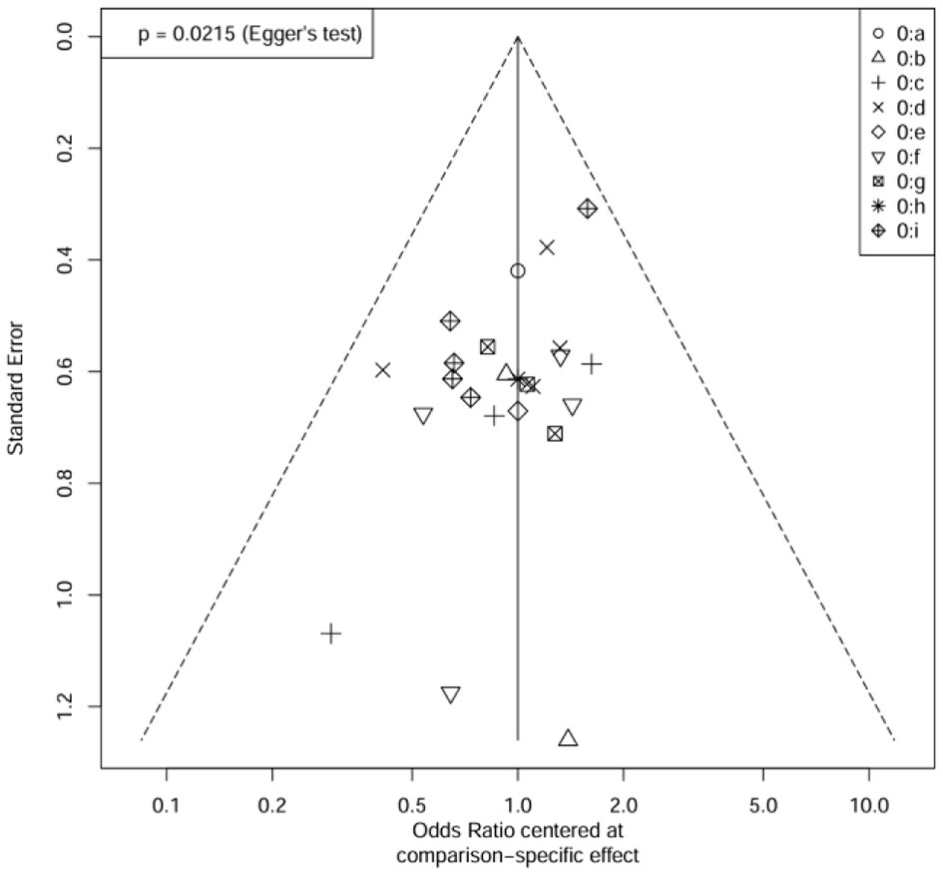
Funnel plot of study effect bias with trim-and-fill analysis.

Visual inspection of the funnel plot revealed a degree of asymmetry, particularly among studies with larger standard errors. This asymmetry was statistically confirmed by Egger's test (*p* = 0.0215). However, the Trim-and-Fill analysis identified no missing studies requiring imputation, suggesting that the pooled effect estimate remained robust despite the observed asymmetry. This discrepancy between Egger's test and the Trim-and-Fill method suggests that the asymmetry may stem from sources other than publication bias, such as between-study heterogeneity or small-study effects, rather than the selective suppression of results. Consequently, the primary conclusions of this meta-analysis appear reliable (Figure 8).

### 3.2 Safety results

The comparative safety assessment, based on CTCAE severity grading and the composite safety score (CSS), is summarized in Table 5. Gastrointestinal and allergic reactions were the most common adverse events, typically mild (CTCAE Grade 1). Xintong Granule presented the most severe risk profile, with a potential for severe (Grade ≥3) hepatotoxicity. Shexiang Tongxin Dropping Pill showed a risk of Grade 1-2 ALT elevation at higher doses. A significant data limitation was the absence of incidence rates for all medications.

**Table 5:** Safety profile and comparative risk assessment of included Chinese patent medicines.

**Medications**	**Adverse reactions & CTCAE Grade Assessment**	**Composite Safety Score (CSS)**
Dengzhan Shengmai Capsule	Gastrointestinal (dry mouth, nausea, distension, diarrhea): Grade 1. Allergic (rash, pruritus): Grade 1. Other (dizziness, palpitations): Grade 1.	A2, B2, C2 = Total: 6
Fufang Dilong Tablet	Gastric discomfort: Grade 1.	A1, B2, C3 = Total: 6
Naoxin Tong Capsule	Uncertain/Not reported (NR).	A3, B1, C1 = Total: 5
Qishen Yiqi Dropping Pill	Gastrointestinal (nausea, vomiting, bloating): Grade 1. Skin/Allergic (rash, pruritus, flushing): Grade 1.	A2, B1, C2 = Total: 5
Shexiang Tongxin Dropping Pill	Body heat/facial flushing: Grade 1. Tingling/numbing tongue: Grade 1. Elevated ALT (higher doses): Grade 1-2 (assuming asymptomatic elevation).	A2, B1, C3 = Total: 6
Tongxinluo Capsule	Gastrointestinal (nausea, vomiting, pain, bloating, diarrhea, discomfort): Grade 1-2. Allergic (rash, pruritus): Grade 1. Dizziness: Grade 1.	A2, B3, C1 = Total: 6
Yangxinshi Tablet	Gastric discomfort: Grade 1.	A1, B1, C1 = Total: 3
Xintong Granule	Hepatobiliary (liver injury): Grade ≥3 (potentially serious). Gastrointestinal (nausea, vomiting, diarrhea, pain, bloating, etc.): Grade 1-2. Skin/Allergic (rash, pruritus, erythema, etc.): Grade 1-2. Other (dizziness, headache, dyspnea): Grade 1-2.	A3, B3, C3 = Total: 9
Qishen Capsule	Uncertain/Not reported (NR).	A3, B3, C3 = Total: 9

**CTCAE Grade Assessment:** Severity is graded per Common Terminology Criteria for Adverse Events (CTCAE) v5.0: Grade 1 (Mild), Grade 2 (Moderate), Grade 3 (Severe), Grade 4 (Life-threatening). Assessment is based solely on qualitative descriptions from drug inserts and included studies; quantitative incidence data were unavailable.

**Composite Safety Score (CSS) Calculation:** The CSS (range: 3-9, lower is better) sums scores from three dimensions derived from the full safety profile:

**A. AE Severity:** 1 = Only Grade 1; 2 = Includes Grade 1-2; 3 = Includes Grade ≥3 risk or **Unknown**.

**B. Contraindications Scope:** 1 = Limited (e.g., pregnancy only); 2 = Moderate (e.g., + specific syndrome/allergy); 3 = Broad (e.g., + hemorrhagic disease, organ injury history) or **Unknown**.

**C. Precautions Complexity:** 1 = Simple (e.g., take with food); 2 = Moderate (e.g., multiple simple items); 3 = Complex (e.g., requires monitoring, dietary/drug restrictions).

**Data Limitation:** The assessment is constrained by the lack of quantitative incidence rates (proportion of affected cases) in source materials.

The Composite Safety Score (CSS) provided an integrated risk ranking. Yangxinshi Tablet had the most favorable profile (CSS = 3), characterized by only mild AEs and straightforward precautions. In contrast, Xintong Granule and Qishen Capsule had the least favorable scores (CSS = 9). Xintong Granule's score was driven by its severe hepatotoxicity risk and complex management requirements, while Qishen Capsule's score primarily reflects critical gaps in reported safety data (AEs and contraindications listed as “Uncertain”). The remaining six CPMs obtained intermediate scores (CSS = 5–6), indicating similar overall risk levels when integrating severity, contraindications, and precautions.

Among the listed medications, gastrointestinal reactions are the most common adverse reactions, primarily manifested as nausea, vomiting, abdominal distension, abdominal pain, and diarrhea. These reactions are associated with medications such as Dengzhan Shengmai Capsule, Qishen Yiqi Dropping Pill, Tongxinluo Capsule, Xintong Granules, and Fufang Dilong Tablet. Six medications carry this risk.

Allergic reactions, including symptoms such as rash, pruritus, flushing, and palpitations, are frequently observed with Dengzhan Shengmai Capsule, Qishen Yiqi Dropping Pill, Shexiang Tongxin Dropping Pill (facial flushing), and Xintong Granule. Patients with allergic predispositions should exercise caution with medications containing insect-derived ingredients (e.g., Earthworm, Leech) or toxic animal components (e.g., Toad venom). Additionally, hepatotoxicity is a severe risk for specific medications, such as Xintong Granule (due to *Polygonum multiflorum*) and Shexiang Tongxin Dropping Pill.

Contraindications in pregnant women are the prevalent, involving six medications: Naoxin Tong Capsule, Shexiang Tongxin Dropping Pill, Tongxinluo Capsule, Yangxinshi Tablet, and Xintong Granule, primarily due to ingredients with blood-activating properties (e.g., Salvia) or toxic components that may cause teratogenicity or miscarriage. Contraindications for hemorrhagic diseases or bleeding risks apply to Dengzhan Shengmai Capsule (acute phase of cerebral hemorrhage), Tongxinluo Capsule (hemorrhagic diseases), and Qishen Yiqi Dropping Pill (when combined with anticoagulants), as their blood-activating and stasis-resolving components may exacerbate bleeding.

Heat-related syndrome contraindications involve Fufang Dilong Tablet (phlegm-heat syndrome, blood stasis-heat syndrome) and Tongxinluo Capsule (Yin deficiency with fire effulgence pattern), requiring strict adherence to TCM syndrome differentiation principles. Contraindications for hepatic or renal impairment target Shexiang Tongxin Dropping Pill and Xintong Granule (hepatotoxicity from *Polygonum multiflorum*) to avoid aggravated metabolic burden or liver injury risks.

### 3.3 Cost analysis results

#### 3.3.1 Medication pricing analysis

Among the nine Chinese patent medicines, Yangxinshi Tablets has the lowest daily treatment cost at CNY 6.24/day, with its cost advantage stemming from a large packaging size (48 tablets/box) and moderate daily dosage (8 tablets/day), effectively reducing per-day medication expenses. In contrast, Qishen Capsule has the highest daily cost (CNY 12.50/day), primarily due to its small packaging (18 capsules/box) and higher daily dosage (9 capsules/day), necessitating frequent purchases and further increasing the financial burden. The daily costs of the remaining medications fall within the range of CNY 6.47–9.60/day, with variations driven by differences in packaging specifications and daily dosages. For example, Xintong Granules (CNY 9.60/day) incurs higher costs due to its small packaging (10 sachets/box) and relatively high daily dosage (4 sachets/day), while Tongxinluo Capsule (CNY 7.50/day) achieves cost balance through medium packaging (40 capsules/box) and moderate dosage (9 capsules/day) (Table 6).

**Table 6:** Medication pricing analysis.

**Medications**	**Price per Box (CNY)**	**Days per Box**	**Daily Cost (CNY)**	**Total Cost (CNY)**	Efficacy Ranking (SUCRA)^*^
Dengzhan Shengmai Capsule	32.58	3 days (18 capsules ÷ 6 capsules/day)	10.86	651.6	88.99%
Fufang Dilong Tablets	44.50	6 days (36 tablets ÷ 6 tablets/day)	7.42	222.5	
Naoxin Tong Capsule	38.81	6 days (72 capsules ÷ 9 capsules/day)	6.47	181.0	
Qishen Yiqi Dropping Pill	33.30	5 days (15 sachets ÷ 3 sachets/day)	6.66	199.8	
Shexiang Tongxin Dropping Pill	43.70	6 days (36 pills ÷ 6 pills/day)	7.28	218.5	75.12%
Tongxinluo Capsule	33.32	4.4 days (40 capsules ÷ 9 capsules/day)	7.50	224.9	
Yangxinshi Tablets	37.44	6 days (48 tablets ÷ 8 tablets/day)	6.24	187.2	42.30%
Xintong Granules	24.00	2.5 days (10 sachets ÷ 4 sachets/day)	9.60	288.0	
Qishen Capsule	25.00	2 days (18 capsules ÷ 9 capsules/day)	12.50	525.0	62.35%

Detailed dosage information is provided in this table. For dosage ranges (e.g., 1-2 sachets/dose, bid-tid), the midpoint value (e.g., 1.5 sachets/dose, 2.5 times a day) is used for calculation. Decimal fractions are uniformly rounded up to the nearest whole unit (e.g., 1 capsule, 1 tablet, 1 sachet). ^*^from Figure 6.

#### 3.3.2 Integrated assessment of costs and efficacy

By synthesizing the DTC (Table 5) with the efficacy rankings (SUCRA), we identified distinct value profiles: Premium Efficacy (High-Cost, High-Benefit): Dengzhan Shengmai Capsule ranked highest in efficacy (SUCRA 88.99%) but also had the second-highest daily cost (CNY 10.86). This option is justified when maximal clinical efficacy is the priority, outweighing economic considerations. Balanced Value Profile (Moderate-Cost, High-Benefit): Shexiang Tongxin Dropping Pill offers the most balanced profile. It achieved the second-highest efficacy (SUCRA 75.12%) while maintaining a moderate daily cost (CNY 7.28). Economic Priority (Low-Cost, Moderate-Benefit): Yangxinshi Tablets is the definitive cost-minimization option (CNY 6.24/day). While its efficacy ranking was lower (SUCRA 42.30%), its favorable safety profile (lowest adverse reactions) and minimal cost make it a strong candidate for long-term use in stable, budget-constrained patients. Low Value (High-Cost, Moderate-Benefit): Qishen Capsule demonstrated a poor lower comparative value, pairing the highest daily cost (CNY 12.50) with only moderate efficacy (SUCRA 62.35%).

#### 3.3.3 Sensitivity analysis of cost parameters

Regarding price stability, all analyzed medications are listed in China's national insurance catalog, where centralized procurement policies maintain relatively stable pricing; thus, price-based sensitivity was assessed as low.

However, sensitivity related to treatment duration and dosage is high. As the inclusion criteria (Table 1) and findings, medications with flexible-dose regimens (e.g., Naoxin Tong Capsule, 2–4 capsules/dose; Xintong Granules, 1–2 sachets/dose) pose significant cost fluctuation risks. A patient requiring the high-dose regimen could see their total medication expenses increase by over 30% compared to the standard-dose calculation used in this analysis, thereby impacting their real-world economic burden.

## 4 Discussion

This study systematically evaluated the multidimensional value of Chinese patent medicines (CPMs) in treating angina pectoris in coronary heart disease (CHD) with Qi deficiency and blood stasis, revealing both their therapeutic potential and existing limitations. The efficacy analysis demonstrated significant advantages of combined CPM-Western medicine regimens, particularly for Dengzhan Shengmai Capsule and Shexiang Tongxin Dropping Pill. The safety evaluation highlighted distinct risk profiles, with Yangxinshi Tablet exhibiting the most favorable profile, while others like Xintong Granule necessitated vigilance regarding hepatotoxicity. The cost analysis identified substantial cost variations, with Yangxinshi Tablet and Fufang Dilong Tablet presenting the lowest financial burden, whereas flexible-dose medications introduced cost uncertainty.

### 4.1 Clinical and practical implications

In clinical practice, applying these findings requires a balanced consideration of efficacy, safety, and economic feasibility. For high-risk populations, such as patients with hepatic impairment, prioritizing safer options like Yangxinshi Tablet is advisable. The management of flexible-dose medications (e.g., Naoxin Tong Capsule) presents a challenge, as current rigid reimbursement policies may not accommodate dose titration, necessitating the development of dynamic reimbursement rules. Furthermore, practical barriers to adherence, such as the long treatment course of Dengzhan Shengmai Capsule (60 days) and the stringent storage conditions (e.g., low-temperature requirements) for some CPMs like Shexiang Tongxin Dropping Pill, must be considered, particularly in resource-limited settings. The correlation between cost and SUCRA efficacy provides a practical guide: Yangxinshi Tablet serves as a cost-effective foundation, while Shexiang Tongxin Dropping Pill offers a balanced high-efficacy option for patients where maximizing symptom control is the priority.

The superior efficacy of CPMs, particularly Dengzhan Shengmai Capsule and Shexiang Tongxin Dropping Pill, when combined with conventional medicine, positions them as a potential complementary strategy within the framework of international guidelines for stable angina management. Current ESC/AHA guidelines (36) recommend a foundation of lifestyle modification and pharmacotherapy, including antiplatelet agents (e.g., aspirin), statins, beta-blockers, calcium channel blockers, and nitrates for symptom control. The findings of this study suggest that the addition of specific CPMs to this standard Western regimen can provide a significant incremental benefit in improving angina symptoms. This aligns with the guideline principle of individualized therapy, offering a structured, evidence-based complementary option, particularly for patients who have a TCM syndrome pattern of Qi deficiency and blood stasis and may experience suboptimal symptom relief with conventional drugs alone. Further high-quality studies directly comparing this integrative approach against guideline-directed intensification of Western therapy are warranted to better define its role in global clinical practice.

### 4.2 Correlation with international integrative cardiology literature

The findings of this study resonate with the growing body of international research on bioactive compounds from traditional medicines for cardiovascular health. The superior performance of Dengzhan Shengmai Capsule, whose main component is *Erigeron breviscapus* (Dengzhan Xixin), aligns with pharmacological studies on its active ingredient, scutellarin, which has demonstrated anti-inflammatory, antioxidant, and endothelial-protective effects in experimental models of ischemia-reperfusion injury (37). Similarly, the efficacy of formulations like Naoxin Tong Capsule and Tongxinluo Capsule, which contain *Salvia miltiorrhiza* (Danshen), is supported by extensive research on its components, tanshinones and salvianolic acids. These compounds have been shown to improve coronary microcirculation, inhibit platelet aggregation, and attenuate atherosclerotic plaque formation—mechanisms that are highly relevant to angina pathophysiology (38). *Panax notoginseng* (Sanqi), a key ingredient in many CPMs including Xintong Granule, is another well-studied herb, with its saponins (*notoginsenosides*) recognized for their hemorheological-improving and cardioprotective properties in systematic reviews (39). Thus, the clinical efficacy observed in this NMA finds a plausible mechanistic basis in modern pharmacological research, bridging TCM clinical practice with global integrative cardiology science.

Furthermore, the multidimensional framework developed in this study demonstrates strong alignment with the core principles of international regulatory science for botanical medicines. For instance, the European Medicines Agency (EMA) assesses herbal medicinal products based on a comprehensive review of quality, safety, and efficacy (40). While our framework is applied specifically to CPMs within the Chinese context, its structured approach—quantifying efficacy via network meta-analysis, systematically profiling safety, and evaluating economic impact—mirrors this tripartite evidence requirement. This methodological congruence suggests that the framework is not merely a local solution, but possesses a foundational architecture that could be adapted by other regulatory and health technology assessment bodies seeking to integrate traditional medicines into mainstream, evidence-based healthcare systems. By providing robust, multi-faceted evidence similar to the requirements of agencies like the EMA or EFSA, this study supports the potential international replicability of TCM evaluation models.

### 4.3 Implications for health policy

The multidimensional profile of these CPMs has direct implications for health policy, particularly in drug access, reimbursement, and post-market surveillance.

Policy interventions, such as centralized procurement for large-packaging drugs, can mitigate economic barriers and improve accessibility. The successful implementation of such policies relies on an efficient healthcare delivery system. As shown by Zhou et al. (41) in their assessment of electronic prescriptions in Chengdu community pharmacies, China is actively modernizing its pharmaceutical care infrastructure to enhance service efficiency and accessibility (41). Building upon such advancements, integrating the multidimensional evaluation framework for CPMs into electronic health record and prescription platforms could support clinical decision-making and streamline the reimbursement process for cost-effective CPMs.

The significant economic disparities and economic burden and value proposition variations among the CPMs argue for a refined approach to their inclusion in national reimbursement drug lists (NRDL). Policymakers could prioritize CPMs with a favorable efficacy-safety-economic profile (e.g., Yangxinshi Tablet, Shexiang Tongxin Dropping Pill) for inclusion or negotiation. Centralized procurement policies should be leveraged to promote large-packaging specifications (e.g., for Naoxin Tong Capsule) to reduce unit costs and minimize packaging waste. For medications with flexible dosing, implementing “dose-linked reimbursement” rules, where the reimbursement level is adjusted based on the prescribed daily dose, could control costs while maintaining prescriber flexibility.

The identification of specific safety signals, such as the hepatotoxicity risk associated with *Polygonum multiflorum* in Xintong Granule, underscores the critical need for a robust and standardized national pharmacovigilance system for CPMs. Mandatory and active post-market safety studies should be enforced, particularly for CPMs with incomplete adverse reaction records, such as Qishen Capsule. Enhancing signal detection also requires the integration of TCM-specific data—including syndrome differentiation and long-term usage patterns—into adverse event reporting platforms. Furthermore, the Common Terminology Criteria for Adverse Events (CTCAE) classification applied in this study could serve as a model for standardizing the severity assessment of CPM-related adverse events in both clinical practice and regulatory reporting.

### 4.4 Methodological limitations and future research

Several methodological limitations of this study warrant careful consideration, as they delineate clear boundaries for interpreting our findings and highlight priorities for future research.

It is worth noting that Shexiang Baoxin Wan, a widely prescribed CPM for stable angina in China, was not included in this network meta-analysis. Although our search strategy identified several RCTs evaluating this medication, they were excluded because they exclusively reported continuous outcome measures (e.g., mean changes in symptom scores or angina frequency) without providing extractable dichotomous responder data (clinical effective rate). Consistent with our statistical analysis plan, we did not convert aggregate continuous data (Mean ± SD) into dichotomous outcomes to ensure the accuracy of the efficacy estimates.

Regarding efficacy, the pairwise meta-analysis exhibited negligible statistical heterogeneity (*I*^2^ = 0%), supporting the use of a fixed-effect model. However, the analysis was constrained by data sparsity within several CPM subgroups. Key interventions such as Dengzhan Shengmai Capsule, Shexiang Tongxin Dropping Pill, and Xintong Granule were each informed by only a single trial, limiting the robustness of their individual point estimates and precluding assessment of within-group consistency. Although the overall class effect remains robust, estimates for specific formulations vary in precision. Notably, potential sources of clinical variation—such as differences in TCM syndrome application or background Western therapies—did not manifest as significant statistical heterogeneity in the pairwise comparisons. Second, potential small-study effects were suggested by funnel plot asymmetry and Egger's test (*p* = 0.0215). A Trim-and-Fill analysis was conducted to evaluate the impact; no imputed studies were required to achieve symmetry, and the adjusted effect estimate remained unchanged from the original (logOR = 4.663, 95% CI: 4.435–4.891). This indicates that while mild asymmetry exists—likely reflecting a predominance of small positive studies—it does not materially alter the primary efficacy conclusions. Third, the generalizability of the economic and accessibility analyses may be limited by data availability, including regional drug pricing variations and incomplete public disclosure of patent information.

While all included trials adhered to standard treatment guidelines by employing consistent therapeutic classes of Western medicines (e.g., antiplatelets, statins, and nitrates), the control groups comprised multi-component regimens with varying specific drug combinations. We acknowledge that this inherent diversity within the ‘Conventional Medicine' comparator is likely a primary contributor to the observed between-study heterogeneity.

A critical appraisal of the included Chinese-language literature is warranted. Although all studies were randomized controlled trials (RCTs), a notable proportion raised some concerns or were at high risk of bias, largely owing to inadequate reporting of allocation concealment and blinding procedures—a known limitation in earlier Chinese clinical research. Nevertheless, sensitivity analyses indicated that the pooled results were robust. Still, the potential for performance and detection bias in these trials necessitates a cautious interpretation of the efficacy estimates. Collectively, these methodological constraints regarding bias and precision suggest that while the overall comparative efficacy is evident, the strength of evidence for specific pairwise comparisons require conservative interpretation.

The safety assessment, while introducing a novel comparative framework, is constrained by the fundamental nature of its source data. The profiles compiled in Table 5 are qualitative, detailing the presence but not the incidence or precise severity of adverse effects. Therefore, while the CTCAE grading and Composite Safety Score provide a valuable standardized comparative framework, they facilitate a hazard identification and relative ranking rather than a quantitative risk estimation. The scores reflect the spectrum and seriousness of potential risks, not their empirically measured frequency. Future high-quality studies that systematically collect and report quantitative safety outcomes are essential to validate and refine these comparative assessments.

Future research should prioritize multicenter, high-quality RCTs with standardized protocols (e.g., for syndrome diagnosis, treatment duration, and outcome measures) to validate the efficacy of high-heterogeneity therapies (e.g., Tongxinluo Capsule) and reduce bias. Long-term, prospective real-world studies integrated with pharmacovigilance data are essential to establish the long-term safety profiles of these CPMs. From a technological and policy perspective, future efforts should focus on optimizing the extraction of toxic components, elucidating multi-target mechanisms through platforms like metabolomics, and establishing dedicated funding for modernizing classical formulas. Internationally, promoting the inclusion of well-evidenced CPMs in global treatment guidelines (e.g., WHO Traditional Medicine Strategy) will be crucial for their rational integration into global cardiovascular care.

## 5 Conclusion

In summary, this study establishes a comprehensive multidimensional framework for evaluating Chinese patent medicines (CPMs) by systematically integrating efficacy, safety, and economic evidence. The results demonstrate that specific CPMs, particularly Dengzhan Shengmai Capsule (for maximal efficacy) and Shexiang Tongxin Dropping Pill (for balanced value), significantly enhance angina relief when adjunctive to standard Western therapy. Conversely, options like Yangxinshi Tablet offer a definitive cost-minimization strategy for budget-constrained settings.

Beyond these specific clinical findings, this study illustrates a rigorous methodological approach for evaluating complex interventions. By aligning with international regulatory standards—specifically mirroring the European Medicines Agency (EMA) requirements for botanical medicines—this structured evaluation model provides a pragmatic tool for health technology assessment and formulary decision-making. This multidimensional framework may serve as a model for future pharmacoeconomic integration of TCM into global cardiovascular care.

## Data Availability

The original contributions presented in the study are included in the article/Supplementary material, further inquiries can be directed to the corresponding authors.
